# Modeling of Concrete Deterioration under External Sulfate Attack and Drying–Wetting Cycles: A Review

**DOI:** 10.3390/ma17133334

**Published:** 2024-07-05

**Authors:** Shanshan Qin, Chuyu Chen, Ming Zhang

**Affiliations:** 1 School of Construction Engineering, Shenzhen Polytechnic University, Shenzhen 518055, China; qinshanshan@szpu.edu.cn; 2 School of Civil and Environmental Engineering, Harbin Institute of Technology, Shenzhen, Shenzhen 518055, China

**Keywords:** concrete, external sulfate attack, drying–wetting cycles, numerical modeling

## Abstract

This paper comprehensively summarizes moisture transport, ion transport, and mechanical damage models applied to concrete under sulfate attack and drying–wetting cycles. It highlights the essential aspects and principles of each model, emphasizing their significance in understanding the movement of moisture and ions, as well as the resulting mechanical damage within the concrete during these degradation processes. The paper critically analyzes the assumptions made in each model, shedding light on their limitations and implications for prediction accuracy. Two primary challenges faced by current models under sulfate attack and drying–wetting cycles are identified: the limited consideration of the coupled effects of chemical and physical attacks from sulfate, and the unclear mechanism of the sulfate attacks. Future research directions are proposed, focusing on exploring the transport mechanism of sulfate ions under various driving forces and further clarifying the crystallization process and expansion damage mechanism in concrete pores. Addressing these research directions will advance our understanding of sulfate attack under drying–wetting cycles, leading to improved models and mitigation strategies for enhancing the durability and performance of concrete structures.

## 1. Introduction

Concrete structures in saline–alkali soils, salt lakes, mining sites, and coastal areas face significant challenges due to the high concentrations of sulfate ions in these regions. The presence of sulfate ions can trigger reactions with the hydration products of concrete, forming expansive products such as ettringite and gypsum. These products exert pressure on the walls of capillary pores within concrete, resulting in cracking, spalling, and softening.

Furthermore, dynamic weather conditions, groundwater fluctuations, and tidal movements introduce drying–wetting cycles to the concrete structures. Extensive studies have demonstrated that exposure to drying–wetting cycles significantly accelerates the degradation rate of concrete compared to full immersion conditions [1,2,3]. During drying–wetting cycles, moisture continuously moves in and out of the concrete, facilitating the penetration of sulfate ions into its inner layers. This increased penetration rate intensifies the potential for sulfate attack. Once sulfate ions infiltrate the concrete, they initiate chemical reactions with the cementitious materials, forming expansive products. Simultaneously, the sulfate ions induce severe physical crystallization damage due to moisture variations. This combined chemical and physical attack accelerates the expansion and cracking of concrete, further exacerbating the degradation of concrete structures [4,5,6].

Extensive research [7,8,9,10,11,12,13,14,15] has been conducted on the degradation of concrete fully submerged in sulfate solutions, providing valuable insights into the influence of sulfate attack on concrete’s mechanical properties. Studies [16,17] have shown that regardless of whether concrete is fully or partially immersed, the volume of pore structures initially decreases during the early stages of sulfate attack. However, in the middle and later stages, the volume increases. This is attributed to the formation of products like chemical reaction products or sodium sulfate crystals, which initially reduce the available pore space. However, as sulfate attack progresses, the destruction of aggregate–paste structures or the precipitation of sodium sulfate crystals leads to the development of cracks, causing an increase in pore volume during the middle and later stages of sulfate attack. The compressive strength, elastic modulus, and tensile strength of concrete under sulfate attack also typically depict a dual-phase trend, characterized by an initial slow increase followed by a significant decrease [18,19,20,21,22]. These changes in mechanical properties can be attributed to the alterations in pore structures caused by sulfate attack. The reduction in pore volume indicates a densification of the concrete, leading to an increase in its mechanical properties. Conversely, when the pore volume increases, the concrete becomes more porous and less dense, resulting in a decrease in its mechanical properties. The expansion trend induced by sulfate attack in concrete is characterized by a slow initial growth, followed by a rapid increase, before finally reaching a stable state [23,24,25,26,27]. This progression occurs as the expansion products gradually fill the pores during the early stage, resulting in a gradual expansion. Once a certain portion of the pores is completely filled, the ongoing increase in expansion products significantly accelerates the expansion rate. When the expansion strain exceeds the tensile strength of the material, cracks are initiated and propagated, providing additional space for further expansion and ultimately leading to a stable state. Furthermore, several studies [28,29,30,31] have proposed that the depth of the sulfate attack can serve as an indicator for assessing concrete degradation. This depth represents the extent of expansion and damage in the affected area and exhibits a similar trend to the expansion behavior.

In order to simulate the damage and deterioration caused by sulfate attack, numerical models have been developed, incorporating processes such as ion transport, chemical reactions, and expansive deformation [7,32,33,34]. However, the process of sulfate attack under drying–wetting cycles presents a significantly more intricate scenario. During the drying–wetting cycles, ion movement in concrete is influenced by diffusion, driven by concentration gradients, and convection, driven by saturation gradients. This combination introduces a more complex ion transport process compared to fully saturated conditions. Both diffusion and convection play significant roles in determining ions’ movement and distribution within the concrete [35]. In drying–wetting cycles, a sulfate attack on concrete involves chemical reactions and physical crystallization. Chemical reactions between sulfate ions and the hydration products of concrete result in the formation of expansive products such as ettringite [5,35,36,37,38,39,40]. Simultaneously, the physical attack occurs as sulfate ions induce crystalline growth due to changes in saturation within the concrete, resulting in expansion and cracking [25,26,41,42,43,44,45,46,47]. However, the precise interplay between these coupled mechanisms during sulfate attack under drying–wetting cycles remains unclear. There is currently a lack of consensus among researchers regarding the specific crystallization processes and types of sulfate within the concrete pores.

Further research is imperative to enhance our understanding of the intricate deterioration process of concrete subjected to sulfate attack and drying–wetting cycles. The present study provides a comprehensive analysis of the numerical modeling of concrete deterioration under sulfate attack and drying–wetting cycles. It offers a systematic overview of the assumptions and limitations associated with the models of moisture transport, ions transport, chemical reaction, physical crystallization, and expansion damage. This analysis serves as a valuable reference for future researchers interested in conducting studies related to deterioration models for concrete under sulfate attack and drying–wetting cycles. By understanding the assumptions and limitations of these models, researchers can build upon existing knowledge and develop more accurate and comprehensive models to better predict the deterioration behavior of concrete under sulfate attack and drying–wetting cycles.

## 2. Moisture Transport Model

Moisture transport within concrete can be effectively described using Darcy’s law and mass conservation principle. These fundamental principles establish a relationship between the flow of moisture through porous media and the permeability coefficients, as follows:

(1) $$\frac{ds}{dx}=D_{m}\left(s\right)\cdot \frac{ds}{dx}$$

where *s* denotes the saturation of pores, which indicates the water content within the concrete. *D_m_* is the moisture transport coefficient. To establish a comprehensive moisture transport model for concrete, defining the moisture transport coefficient is crucial to establish a relationship between the moisture content and the pressure gradient. This relationship serves as a key component in understanding how pressure changes influence moisture movement within the concrete. Several researchers have proposed different models for moisture transport in concrete based on various assumptions. These models can be broadly categorized into two main types: theoretical models and empirical models.

### 2.1. Theoretical Model

Theoretical models are based on fundamental principles and mathematical equations derived from the governing equations of fluid flow and transport phenomena. These models aim to describe the physical processes and mechanisms involved in moisture transport within the concrete matrix. 

Under drying–wetting cycles, the moisture state within concrete undergoes continuous changes. Pel et al. [48] observed that concrete’s water vapor transport coefficient varies with internal saturation levels. Their findings indicate that the coefficient increases with higher internal saturation levels and decreases with increasing saturation at lower levels. This behavior can be attributed to the change in the primary form of water transport within the concrete. In highly saturated conditions, liquid water becomes the dominant mode of water transport. As the amount of liquid water increases, water transfer efficiency also increases, resulting in a higher water vapor transport coefficient. However, as the saturation level falls below a critical threshold, gaseous water vapor replaces liquid water as the primary mode of transport. In this case, water vapor density increases as saturation decreases. This increase in water vapor density leads to faster water vapor transport through the concrete. To capture the water transport phenomenon under drying–wetting cycles, Zhang et al. [49] proposed a model that considers two components of water within the concrete: gaseous water vapor and liquid water. They separately investigated the contributions of these two states to the water vapor transport coefficient, as represented by Equation (2).

(2) $$D_{m}\left(s\right)=D_{l}\left(s\right)+D_{v}\left(s\right)=\left(k_{l}+k_{v}\right)\frac{\partial p}{\partial s}$$

where *D_l_* and *D_v_* represent the transport coefficients of liquid water and water vapor, respectively. *k_l_* and *k_v_* indicate the permeability coefficients of liquid water and water vapor, respectively. *s* denotes the saturation of pores within the concrete, indicating the water content. *p* is the pore pressure, which is associated with relative humidity (RH) and represents the energy state of water within the concrete [50].

**Figure 1:**
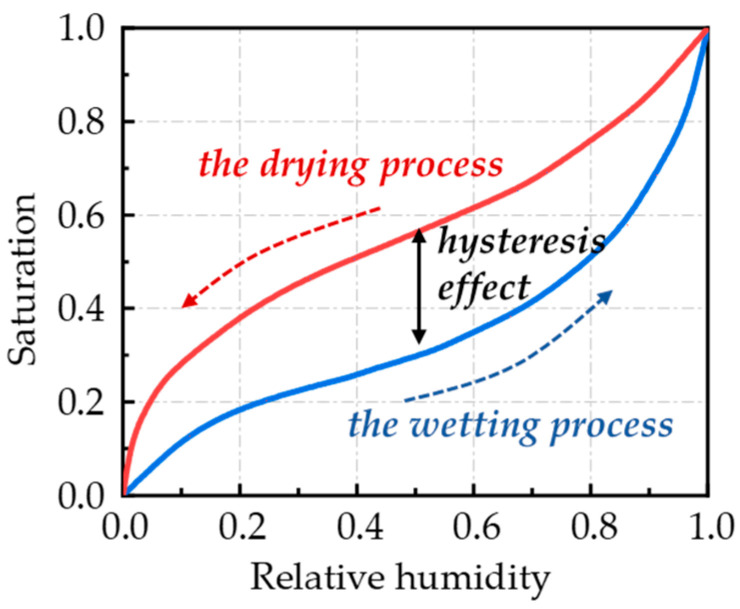
The relationship between saturation and relative humidity in isothermal adsorption.

The balance in the relationship between the moisture content (*s*) within concrete and the energy (*p*) or relative humidity (RH) is a crucial aspect in the study of moisture migration within concrete. The moisture transport process within concrete exhibits a hysteresis effect, as depicted in Figure 1. During the drying process, as the RH decreases, the moisture within the larger pores tends to evaporate first. However, as the RH drops further, the moisture in the smaller pores may become trapped and unable to freely evaporate due to the blockage of the evaporative pathway by the surrounding smaller pores. This hindering of moisture movement results in the drying curve in the relationship between moisture content (*s*) and RH being higher than the wetting curve. In other words, for a given RH, the concrete will have a higher moisture content during the drying stage compared to the wetting stage. To account for the hysteresis effect, Zhang et al. [49] developed separate equations for moisture content during the drying and wetting processes, as shown in Equations (3) and (4):

Drying process:

(3) $$s_{d}=1-\exp(-B\cdot r_{c})$$

**Figure 2:**
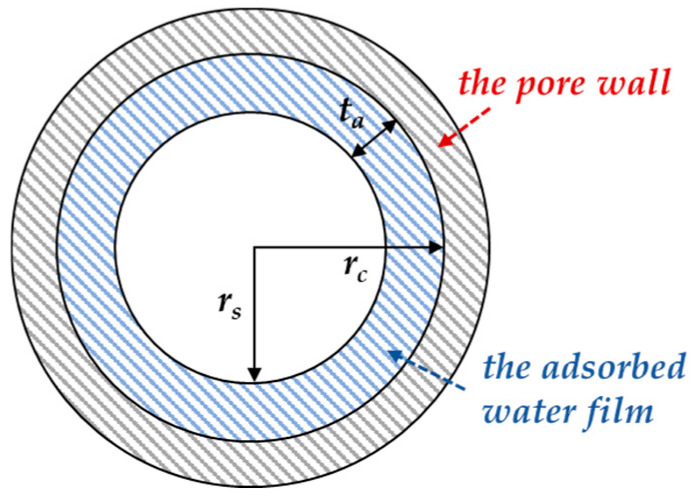
Schematic diagram of modified critical pore radius (*r_s_* is the ideal critical pore radius; *t_a_* is thickness of the adsorbed water film).

Wetting process:

(4) $$s_{w}=\left[1-\exp(-B\cdot r_{c})\right]\times \left[1-\ln\left(1-\exp(-B\cdot r_{c})\right)\right]$$

where *B* represents the parameter of the Rayleigh–Ritz distribution (R-R distribution), which can be obtained from mercury intrusion porosimetry tests. *r_c_* denotes the modified critical pore radius, taking into account the adsorbed water film on the surface of the pores, which is adsorbed by the pore solution, as shown in Figure 2. The relationship between the modified critical pore radius (*r_c_*) and the pore pressure (*p*) can be expressed as follows:

(5) $$r_{c}=\frac{2C\gamma \cos\theta }{p}$$

where *C* represents the regression parameter, based on the modified BET (Brunauer–Emmett–Teller) theory [51], taking 2.15. *γ* is the liquid surface tension, with a value of 0.073 N/m. *θ* denotes the contact angle between water and cement, assuming complete wetting of concrete, so *θ* is 0. By substituting Equation (5) into Equations (3) and (4), the relationship between moisture content (*s*) and pore pressure (*p*) is established as follows:

Drying process:

(6) $$s_{d}=\left[1-\exp\left(\frac{2BC\gamma \cos\theta }{p}\right)\right]$$

Wetting process:

(7) $$s_{w}=\left[1-\exp\left(\frac{2BC\gamma \cos\theta }{p}\right)\right]\cdot \left[1-\ln\left(1-\exp\left(\frac{2BC\gamma \cos\theta }{p}\right)\right)\right]$$

For the permeability coefficients of liquid water (*k_l_*) in Equation (2), assuming that the capillary pores within the concrete are all interconnected cylindrical pores, and the convective flow of the pore solution occurs only within saturated pores while neglecting the transport of water vapor in unsaturated pores, then the coefficient *k_l_* can be simplified as [52]:

(8) $$k_{l}=\frac{\rho_{l}\varphi^{2}}{50B^{2}\eta }{\left(1-\left(1-\frac{2BC\gamma \cos(\theta )}{p}\right)\cdot \exp(2BC\gamma \cos(\theta )/p)\right)}^{2}$$

where *ρ_l_* represents the density of liquid water (kg/m^3^). *φ* is the porosity of concrete. *η* is the viscosity coefficient of the pore solution (Pa·s). 

Different researchers have proposed various models for the permeability coefficients of water vapor (*k_v_*) in Equation (2). Zhang [49] developed a calculation model for the saturation degree (s) and the relative humidity (RH) within the concrete, expressed as follows:

(9) $$k_{v1}=\frac{\rho_{v}\varphi D_{v}}{{\left(\pi /2\right)}^{2}}\frac{1-s}{1+l_{m}/2\left(r_{m}-t_{m}\right)}\left(\frac{M_{w}}{\rho_{l}RT}\right)\exp^{2}\left(\frac{pM_{w}}{\rho_{l}RT}\right)$$

where *ρ_v_* represents the density of water vapor(kg/m^3^). *D_v_* is the free diffusion coefficient of water vapor (m^2^/s). *l_m_* denotes the mean free path of water vapor molecules (m). *r_m_* represents the average radius of unsaturated pores (m). *t_m_* is the thickness of the adsorbed liquid water layer in a pore of size *r_m_* (m). *M_w_* indicates the molar mass of water (18 g/mol). *R* is the universal gas constant (8.3143 J/mol/K). *T* represents the temperature (K). It is noted that this equation encompasses many parameters and is complex to solve. To simplify it, Wu et al. [53] adopted the equation of *k_v_*_2_ with relative humidity as a variable, as follows:

(10) $$k_{v2}=D_{v}{\left[1+\frac{{\left(1+\mathrm{RH}\right)}^{4}}{{\left(1+{\mathrm{RH}}_{0}\right)}^{4}}\right]}^{-1}\frac{\partial \left(\mathrm{RH}\right)}{\partial p}$$

where RH_0_ represents the critical relative humidity (75%). However, in the moisture transport models, the variable is generally the moisture content (*s*). Sun et al. [35], considering the influence of porosity and temperature, provided a relationship between moisture content (*s*) and permeability coefficients of water vapor (*k_v_*_3_) as follows:

(11) $$k_{v3}=0.217\frac{p_{atm}}{p_{g}}{\left(\frac{T}{T_{0}}\right)}^{1.88}\varphi^{a}{\left(1-s\right)}^{b}\frac{\rho_{v}M_{w}}{\rho_{l}^{2}RT\varphi }$$

where *P_atm_* is the reference atmospheric pressure (Pa). *P_g_* represents the vapor pressure (Pa). *T*_0_ denotes the reference temperature (K). *a* and *b* are the fitting parameters. 

After establishing the relationship between moisture content (*s*) and pore pressure (*p*) and determining the parameters for permeability coefficients of liquid water (*k_l_*) and water vapor (*k_v_*), the moisture transport coefficient models for both drying and wetting conditions can be obtained by substituting these parameters into Equation (1). This model is developed based on an understanding of the internal moisture migration mechanism within concrete, considering the separate transport of gaseous water vapor and liquid water to accurately represent the drying and wetting processes. By utilizing this model, researchers can better understand the complex moisture transport phenomena occurring within concrete. This includes the pore structure, saturation levels, and the hysteresis effect observed between drying and wetting. The model provides insights into how different parameters influence moisture transport and helps predict moisture content under various conditions. While the theoretical model for moisture transport in concrete offers a comprehensive understanding of the underlying mechanisms, it can present challenges when applied in practical engineering applications. The model equations can be complex, involving numerous parameters. This complexity can make implementation challenging, both in terms of computational feasibility and obtaining accurate parameter values.

### 2.2. Empirical Model

Empirical models are constructed by analyzing experimental observations and data. These models use statistical correlations and regression analyses to establish relationships between input parameters and moisture transport behavior. Empirical models are simpler to implement and require fewer input parameters compared to theoretical models. Table 1 presents empirical models for moisture transport coefficients during the drying and wetting processes obtained by different researchers.

Li et al. [50] utilized theoretical and empirical models for moisture transport coefficients to predict water loss and absorption in concrete during the drying and wetting processes. These models were compared with the experimental results to assess their accuracy in capturing moisture transport behavior. The findings indicated that both the theoretical and empirical models effectively predict concrete moisture transport. However, the empirical model demonstrated greater accuracy in predicting moisture distribution during wetting. Similarly, Sun et al. [35] conducted experiments to investigate moisture transport in concrete and compared the empirical model to the experimental data. Their results showed a close alignment between the empirical model and the experimental data, particularly during the drying process. This close agreement between the empirical model and the experimental results further supports the effectiveness of the empirical approach in capturing moisture transport behavior in concrete. 

In summary, both empirical and theoretical models for moisture transport coefficients can effectively capture the moisture transport behavior in concrete during drying and wetting processes. However, empirical models have distinct advantages in practical applications. Empirical models demonstrate a good fitting performance, accurately representing the moisture transport behavior observed in the experimental data. They are user-friendly and easy to use, requiring minimal theoretical knowledge for implementation. Additionally, empirical models offer fast calculations, enabling efficient assessments and prompt decision-making. Due to their simplicity, accuracy, and efficiency, numerous researchers favor empirical models for practical applications. As for the modeling approach, empirical models often employ an exponential relationship to describe moisture transport during wetting. This relationship captures the non-linear nature of moisture absorption and adsorption phenomena during wetting. During drying processes, the moisture transport coefficient is often assumed to be constant in empirical models. This assumption simplifies the modeling process and provides a reasonable approximation for drying conditions where the moisture content decreases.

## 3. Ion Transport Model

Under saturated conditions, the transport model for sulfate ions in concrete is typically based on Fick’s law, which describes diffusion as the predominant mechanism for ion transport. However, under conditions of drying–wetting cycles, the driving force for ion transport changes, and the convective effect becomes influential [37]. The convective effect refers to the movement of ions through the concrete due to the flow of water, which is particularly important in the surface layer of concrete subjected to drying–wetting cycles. This highlights the need to consider diffusion and convection mechanisms in the transport model to predict ion ingress and assess ion-induced damage in concrete accurately.

Liu et al. [59] established a transport model for sulfate ions under drying–wetting cycles, assuming that the amount of ion substance entering the concrete during each wetting process is proportional to the moisture content. After *k* drying–wetting cycles, the amount of sulfate ion substance in the pore solution, denoted as *n^k^*, equals the following:

(12) $$n^{k}=\left\{\begin{array}{ll} n^{k-1}+c_{p}v_{p}\Delta s, & the\;wetting\;process\\ n^{k-1}, & the\;drying\;process \end{array}\right.$$

where the *c_p_* is the concentration of sulfate ions in the pore solution (mol/m^3^), *v_p_* is the volume of pores in the concrete (m^3^), and Δ*s* is the difference in the saturation degree of the pore solution between the initial and subsequent moments. In the described model, the total amount of sulfate ion is assumed to remain constant during the drying process. As moisture evaporates from the concrete, the pore solution volume decreases, increasing the concentration of sulfate ions. This change in pore solution concentration reflects the evolving characteristics of sulfate ion concentration throughout the drying process. However, this assumption oversimplifies the situation by neglecting ion diffusion and the consumption of sulfate ions through chemical reactions with cementitious materials. As a result, the model implies that sulfate ion levels in the concrete only increase with the number of drying–wetting cycles, disregarding the potential depletion of sulfate ion concentration caused by chemical reactions. 

Zheng et al. [60] developed a model based on Fick’s law to investigate the transport of sulfate ions in concrete. They incorporated alternating ion concentration boundary conditions to account for the influence of drying–wetting cycles on sulfate ion transport. The formulation of their model is as follows:

(13) $$\frac{\partial c_{SO}}{\partial t}=\frac{\partial }{\partial x}\left(\left(\varphi \cdot D_{SO}\right)\frac{\partial c_{SO}}{\partial x}\right)-k\cdot c_{SO}\cdot c_{CA}$$

where *c_SO_* and *c_CA_* represent the concentration of sulfate ions and calcium aluminates in the matrix (mol/m^3^), respectively. *D*_SO_ is the diffusion coefficient of sulfate ions (m^2^/s). *k* denotes the chemical reaction rate constant (m^3^/mol/s). *t* indicates the diffusion time. By considering these varying conditions, we aimed to enhance the understanding of how sulfate ions are transported in concrete under different moisture conditions. However, it should be noted that this model does not account for factors such as moisture content and convection. 

Zhang et al. [61] introduced a connection between moisture transport and sulfate diffusion in their model by incorporating the saturation degree. They accounted for the hysteresis effect of pore solution evaporation and changes in porosity due to chemical reactions. The ion transport equation is expressed as follows:

(14) $$\frac{\partial c_{SO}}{\partial t}=\mathrm{div}(D_{SO}\cdot \varphi \cdot s\cdot \nabla c_{SO})+\frac{\partial c_{d}}{\partial t}$$

(15) $$\frac{\partial (\varphi s)}{\partial t}=\mathrm{div}\left[D_{md}(\varphi ,s)\cdot \nabla (\varphi s)\right]$$

(16) $$\frac{\partial (\varphi s)}{\partial t}=\mathrm{div}\left[D_{mw}(\varphi ,s)\cdot \nabla (\varphi s)\right]$$

where *c_d_* is the concentration of sulfate ions consumed by chemical reactions with cement materials. *D_md_* and *D_mw_* indicate the moisture transport coefficient *D_m_* during the drying and wetting processes, respectively. On this basis, Li et al. [62] considered convection as follows:

(17) $$\frac{\partial c_{SO}}{\partial t}=\frac{1}{r}\frac{\partial }{\partial r}(D_{SO}\cdot s\cdot \nabla c_{SO}+D_{m}(s)\nabla (s))-\frac{\partial c_{d}}{\partial t}$$

where *r* is the radial distance. This comprehensive model considers various factors influencing ion transport, including moisture transport, chemical reactions, and convective effects. 

Shan et al. [63] considered diffusion, convection, and chemical reactions in their model for sulfate ion transport under drying–wetting cycles. They incorporated factors such as porosity, tortuosity, sulfate attack-induced damage, and humidity in the diffusivity of sulfate ions. The model is expressed as follows:

(18) $$\frac{\partial \left(\varphi sc_{SO}\right)}{\partial t}+\nabla \left(-D_{SO}\nabla c_{SO}\right)+\varphi sc_{SO}\frac{\partial s}{\partial t}+\frac{\partial \left(\varphi sc_{d}\right)}{\partial t}=0$$

(19) $$D_{SO}=f_{d}\left(d_{c}\right)f_{1}\left(s\right)\frac{\varphi }{\tau }D_{s0}$$

where *D_s_*_0_ is the diffusion coefficient of sulfate ions in water. *τ* is the tortuosity. *f_d_*(*d_c_*) and *f*_1_(*s*) represent the influence of sulfate attack-induced damage and pore moisture content on diffusion coefficient, respectively. Additionally, Yin et al. [57] extended the model by including the influence of temperature on the diffusion coefficient, expressed as follows:

(20) $$f_{T}\left(T\right)=\exp\left(2170\left(\frac{1}{T_{ref}}-\frac{1}{T}\right)\right)$$

where *T_ref_* is the reference temperature.

These sulfate transport models highlight the importance of considering multiple factors, such as diffusion, convection, chemical reactions, moisture content, porosity, and temperature, to accurately predict sulfate ion transport and assess its impact on concrete structures. By incorporating these factors, researchers strive to develop comprehensive models that capture the complexities of sulfate ion transport phenomena and improve our understanding of sulfate attack mechanisms in concrete.

## 4. Expansion Damage Mechanism

### 4.1. Microscopic Expansion Mechanisms

The mechanism of concrete damage caused by sulfate attack continues to be a topic of substantial debate among researchers [5,36]. Despite numerous studies and research efforts, there is still no consensus on the exact mechanisms underlying sulfate attack on concrete. The most widely applied theories in the field are the volume increase theory and the salt crystallization theory. The volume increase theory suggests that during the process of a sulfate attack, the generation of expansive products leads to an additional increase in volume, resulting in expansion. This theory quantifies the expansion by calculating the difference in volume between the solid reaction products and the solid reactants:

(21) $$\nu =\frac{\sum V_{s}-\sum V_{r}}{\sum V_{r}}$$

where *ν* is the expansion coefficient. ∑*V_s_* is the total volume of solid reaction products. ∑*V_r_* is the total volume of solid reactants. The volume increase theory provides a quantitative method for assessing the volumetric changes during a sulfate attack on concrete. It establishes a direct relationship between the macroscopic expansion of concrete and the amount of expansion products formed [27]. This approach simplifies the calculation by focusing solely on the volume occupied by the solid reactants and products. However, it is crucial to acknowledge that this method lacks adequate experimental evidence to substantiate its accuracy [25,36,64]. In addition, thermodynamic calculations have revealed that when considering water consumption, the total volume of reactants can be smaller than the total volume of products [27]. Some researchers have also pointed out that the additional volume formed by ettringite and gypsum is typically lower than the available free pore space [65]. These findings highlight the importance of considering factors such as water consumption and available pore space when evaluating the volume changes associated with sulfate attack.

The salt crystallization theory suggests that the expansion observed in concrete is attributed to the crystallization of sulfates from supersaturated solutions. This crystallization process exerts pressure on the pore walls, expressed as follows:

(22) $$P=\frac{RT}{V_{cry}}\ln\left(\frac{Q}{K}\right)$$

where *P* is pressure (Pa). *V_cry_* represents the molar volume of the crystal (m^3^/mol). *K* indicates the equilibrium constant. *Q* is the ionic activity product. In the given equation, the generation of salt crystallization pressure requires the fulfillment of two necessary conditions. Firstly, the ionic activity product must be larger than the equilibrium constant, where *Q* > *K*; otherwise, the calculated salt crystallization pressure would be zero or negative. Secondly, the growing crystals must be restricted within the confined space of the concrete pores. As they continue to crystallize, the crystals exert pressure against the pore walls, causing them to expand. This is why not all formed crystals exhibit expansion, as the crystals need to be constrained to generate crystallization pressure. Some researchers have assessed the influence of crystal size and shape on salt crystallization pressure under constrained conditions. Scherer et al. [41,42,43,66] demonstrated an inverse relationship between the pressure exerted and the size of the pore. It is suggested that smaller pores may experience higher pressure from salt crystallization. Some researchers [42,43,64,67] thought that the morphology of crystals determines the energy balance between the surface atoms of the crystal and the concentration of ions in the surrounding solution. Smaller crystals with higher curvature can generate greater crystallization pressure in equilibrium conditions. Accordingly, the locations of ettringite growth within the microstructure have a greater influence on the development of expansive forces during sulfate attack, rather than the total volume of ettringite formed [5]. The crystallization pressure theory has been extensively discussed from a theoretical perspective in various publications, and it is widely recognized as the most plausible explanation. However, researchers have faced challenges in obtaining conclusive experimental data to directly support this theory in the context of sulfate attack [25].

Bary et al. [46] introduced a chemo–transport–mechanical model, proposing that the observed expansion during sulfate attack is a consequence of the combined effects of volume increase and salt crystallization. They found that the calculated free expansion rate aligns closely with experimental measurements. Furthermore, their research suggests that the contribution of crystallization pressure can be neglected when compared to the strain induced by the increased volume resulting from ettringite formation. This indicates that volume increase plays a dominant role in macroscopic strain development. Ikumi et al. [26] also pointed out that the volume increase and salt crystallization theories may be compatible, as they represent two different sulfate attack stages. According to their perspective, when sulfate ions enter and form ettringite, eventually reaching the solubility limit, the system tends to restore equilibrium via precipitating ettringite. However, if the release of energy through crystal precipitation is hindered, it is released as pressure on the pore walls, resulting in the formation of microcracks. These microcracks alleviate the pressure conditions within the pores, leading to ettringite precipitation near the cracks. Consequently, a proportional increase in the macroscopic free strain corresponds to the amount of precipitated ettringite. This suggests that the initial generation of macroscopic strain is primarily attributed to the action of crystallization pressure, while macroscopic free expansion is explained by a volume increase. 

### 4.2. Macroscopic Expansion Calculation

Numerous scholars have employed a variety of theories to conduct numerical simulations of the mechanical degradation and damage process of concrete induced by sulfate attack and drying–wetting cycles. Chen [68] presented a methodology that utilizes crystallization pressure and micropore mechanics to convert the internal crystallization pressure into strains within concrete. The approach can be summarized as follows:

(23) $$\varepsilon =\left[\left(P_{l}+S_{c}\frac{RT}{V_{m}}\ln\beta \right)\times v_{p}\times \left(\frac{V_{a}}{V_{c}}\right)\right]/k_{e}$$

where *ε* represents the strain in concrete. *P_l_* denotes the pressure generated by the salt solution (MPa). *k_e_* is the elastic modulus after a certain period of degradation (MPa). *S_c_* indicates the volume fraction of crystals. *V_m_* stands for the molar volume of the crystals. *β* is the supersaturation of the pore solution (cm^3^/mol). *V_a_*/*V_c_* represents the volume fraction of aggregates. *v_p_* is the coefficient that accounts for the influence of pores. In this model, the relationship between physical crystallization stress and ion concentration in concrete under drying–wetting cycles is established using sodium nitrate, while the damage caused by chemical attack is determined through full immersion with sodium sulfate. The physical and chemical stress obtained from these experiments are combined to calculate the total erosion strain caused by sulfate attack under drying–wetting cycles. The assumption underlying this approach is that, under identical concentration and wet–dry cycle conditions, the ion concentration distribution and physical crystallization stress generated by sodium nitrate are equivalent to those of sodium sulfate. However, it is important to note that the crystallization pressure of sodium nitrate is significantly lower than that of sodium sulfate.

Yang [69] assumed that the precipitation of Na_2_SO_4_·10H_2_O during the wetting process is the primary cause of physical crystallization damage. Based on the theory of porous media mechanics, the strains generated in concrete during the wetting process were characterized as follows:

(24) $$\varepsilon =\frac{bp_{l}+b_{c}\frac{RT}{V_{m}}\ln\beta }{K}$$

where *K* represents the bulk modulus of concrete (MPa). *b* and *b_c_* denote the Biot coefficient of concrete and crystals, respectively. However, this model overlooks the influence of sulfate ions reacting with cementitious materials. The formation of expansive products can lead to additional internal stresses and damage within the concrete, significantly impacting the mechanical degradation and damage process. 

Ren et al. [70] developed a numerical model that considers both the chemical and physical effects of sulfate attack under drying–wetting cycles. By analyzing the volume fractions of CaSO_4_·2H_2_O and Na_2_SO_4_·10H_2_O within the pores following various numbers of drying–wetting cycles, the macroscopic tensile stress *P*_C_ is calculated as follows:

(25) $$P_{C}=\frac{RT}{v_{M}}\left(\ln K_{SP,T}-\ln K_{SP,M}+10\ln\left(\frac{RH_{sat,T}}{100}\right)\right)+\frac{RT}{v_{G}}\ln\left(\frac{Q_{reac\;}}{K_{reac\;}}\right)$$

where *RH_sat,T_* represents the relative humidity. *K_SP,i_* is the thermodynamic solubility product for the corresponding crystal *i*. *Q_reac_* denotes the ionic activity of the reactants. *K_reac_* indicates the chemical reaction equilibrium constant. In this model, only the formation of gypsum during the chemical reaction is considered, and a linear increase in the concentration of sulfate ions within the concrete is assumed, starting from an initial concentration. However, this assumption contradicts the actual ion transport behavior observed in the process [71]. 

## 5. Future Work

The aforementioned works provide a comprehensive analysis of numerical models, including the moisture transport model, ion transport model, and mechanical damage model. These models are specifically designed to assess the degradation of concrete under the conditions of sulfate attack and drying–wetting cycles. Overall, the current models for sulfate attack under drying–wetting cycles face two primary challenges. Firstly, many existing mechanical damage models tend to linearly combine the effects of sulfate chemical attacks and physical attacks, overlooking a comprehensive consideration of their coupled effects. Secondly, the mechanism of sulfate attack under drying–wetting cycles remains unclear.

To address these limitations, exploring the transport mechanism of sulfate ions within concrete under various driving forces, including concentration gradient, saturation gradients, calcium leaching, temperature effects, and loading effects, is crucial for gaining valuable insights into the deterioration process. Investigating these factors, either individually or in combination, will enhance our understanding of how sulfate ions migrate and interact within the concrete matrix. This can help to identify the pathways and mechanisms through which sulfates penetrate the concrete and initiate the degradation process. 

Moreover, it is advisable to conduct experimental investigations to separately clarify the effects of sulfate chemical attack, physical attack, and the coupled chemical–physical attack on the expansion and mechanical properties degradation of concrete. Microscopic analysis techniques such as Scanning Electron Microscopy (SEM), X-ray Diffraction (XRD), Mercury Intrusion Porosimetry (MIP), and Thermogravimetric Analysis (TGA) can be employed to observe and analyze the microstructural changes, the pore structures, and the formation of expansive products during the attacking process. Establishing an experimental relationship between expansion and microstructural evolution can help to reveal the degradation mechanism of different sulfate attack modes at macro- and micro-levels. This relationship can help to clarify the crystallization process and expansion damage mechanism of sulfates in concrete pores, and help to understand the contributions of both sulfate chemical and physical attacks.

Furthermore, once the relationship between sulfate attack and the mechanical degradation of concrete is established, it becomes possible to develop a mechanical degradation model specifically tailored to sulfate-attacked concrete. This model can simulate the deterioration process of concrete by incorporating approaches such as smeared crack approaches, cohesive zone approaches, and the moving mesh technique [72,73]. These methods allow for the simulation of crack propagation and the associated mechanical damage caused by sulfate attack.

Finally, conducting a study on the durability failure of sulfate-attacked concrete would be valuable. This study aims to predict the lifespan of concrete structures under sulfate attack. By integrating the mechanical degradation model with other factors, such as environmental conditions, loading conditions, and structural design, researchers can assess the long-term durability and performance of concrete structures affected by sulfate attack. This prediction of the durability lifespan can guide decision-making processes for maintenance, repair, and replacement strategies, ultimately ensuring the longevity and safety of concrete structures in real-world construction scenarios.

## 6. Conclusions

This paper offers a comprehensive summary of the moisture transport, ion transport, and mechanical damage models applied to concrete under sulfate attack and drying–wetting cycles. It provides an overview of the essential aspects and principles of each model, emphasizing their significance in comprehending the movement of moisture and ions, along with the consequent mechanical damage within the concrete caused by these degradation processes. This paper also critically analyzes the assumptions made in each model, shedding light on their limitations and the potential implications for the accuracy of the predictions. In addition, the paper suggests future research directions to further enhance our understanding of sulfate attacks on concrete. One important area of focus is the exploration of the transport mechanism of sulfate ions under various driving forces. Another is the need to clarify the crystallization process and expansion damage mechanism within concrete pores and to establish an experimental relationship between expansion and microstructural evolution to reveal the degradation mechanism at both the macro- and micro-levels. By addressing these research directions, future studies can contribute to advancing our knowledge of sulfate attack under drying–wetting cycles. This will lead to the development of more accurate and comprehensive models that capture the complex interactions and mechanisms involved in sulfate attack-induced damage. Ultimately, this research will aid in the development of improved mitigation strategies to enhance the durability and performance of concrete structures.

## Figures and Tables

**Table 1:** Empirical models for moisture transport coefficient.

Stage	Function Form	Expression	Reference
Wetting	Exponential	$D_{m}=D_{m0}\cdot e^{ns}$	Li [54], Guan [55], Gummerson [56]
	Power function	$D_{m}=D_{m0}\cdot s^{n}$	Yin [57]
Drying	S-shaped	$D_{m}=D_{m0}\left[\alpha_{1}+\frac{1-\alpha_{1}}{1+{\left((1-s)/\left(1-s_{c}\right)\right)}^{N}}\right]$	Li [54], Guan [55], Wong [58]
	Constant	$D_{m}=D_{1}$	Li [50]
